# A repeated measures study of phenol, paraben and Triclocarban urinary biomarkers and circulating maternal hormones during gestation in the Puerto Rico PROTECT cohort

**DOI:** 10.1186/s12940-019-0459-5

**Published:** 2019-04-02

**Authors:** Amira M. Aker, Kelly K. Ferguson, Zaira Y. Rosario, Bhramar Mukherjee, Akram N. Alshawabkeh, Antonia M. Calafat, José F. Cordero, John D. Meeker

**Affiliations:** 1 Department of Environmental Health Sciences, University of Michigan School of Public Health, Room 1835 SPH I, 1415 Washington Heights, Ann Arbor, MI 48109-2029 USA; 2 Epidemiology Branch, Intramural Research Program, National Institute of Environmental Health Sciences, Durham, USA; 3 Graduate School of Public Health, Medical Sciences Campus, University of Puerto Rico, San Juan, PR USA; 4 Department of Biostatistics, University of Michigan School of Public Health, Ann Arbor, MI USA; 5 College of Engineering, Northeastern University, Boston, MA USA; 6 Centers for Disease Control and Prevention, Atlanta, GA USA; 7 College of Public Health, University of Georgia, Athens, GA USA

**Keywords:** Thyroid hormones, Reproductive hormones, Pregnancy, In-utero, Endocrine disruption, Phenols, Parabens, Triclocarban

## Abstract

**Introduction:**

Prenatal exposure to some phenols and parabens has been associated with adverse birth outcomes. Hormones may play an intermediate role between phenols and adverse outcomes. We examined the associations of phenol and paraben exposures with maternal reproductive and thyroid hormones in 602 pregnant women in Puerto Rico. Urinary triclocarban, phenol and paraben biomarkers, and serum hormones (estriol, progesterone, testosterone, sex-hormone-binding globulin (SHBG), corticotropin-releasing hormone (CRH), total triiodothyronine (T3), total thyroxine (T4), free thyroxine (FT4) and thyroid-stimulating hormone (TSH)) were measured at two visits during pregnancy.

**Methods:**

Linear mixed models with a random intercept were constructed to examine the associations between hormones and urinary biomarkers. Results were additionally stratified by study visit. Results were transformed to hormone percent changes for an inter-quartile-range difference in exposure biomarker concentrations (%Δ).

**Results:**

Bisphenol-S was associated with a decrease in CRH [(%Δ -11.35; 95% CI: -18.71, − 3.33), and bisphenol-F was associated with an increase in FT4 (%Δ: 2.76; 95% CI: 0.29, 5.22). Butyl-, methyl- and propylparaben were associated with decreases in SHBG [(%Δ: -5.27; 95% CI: -9.4, − 1.14); (%Δ: -3.53; 95% CI: -7.37, 0.31); (%Δ: -3.74; 95% CI: -7.76, 0.27)]. Triclocarban was positively associated with T3 (%Δ: 4.08; 95% CI: 1.18, 6.98) and T3/T4 ratio (%Δ: 4.67; 95% CI: -1.37, 6.65), and suggestively negatively associated with TSH (%Δ: -10.12; 95% CI: -19.47, 0.32). There was evidence of susceptible windows of vulnerability for some associations. At 24–28 weeks gestation, there was a positive association between 2,4-dichlorophenol and CRH (%Δ: 9.66; 95% CI: 0.67, 19.45) and between triclosan and estriol (%Δ: 13.17; 95% CI: 2.34, 25.2); and a negative association between triclocarban and SHBG (%Δ: -9.71; 95% CI:-19.1, − 0.27) and between bisphenol A and testosterone (%Δ: -17.37; 95% CI: -26.7, − 6.87).

**Conclusion:**

Phenols and parabens are associated with hormone levels during pregnancy. Further studies are required to substantiate these findings.

**Electronic supplementary material:**

The online version of this article (10.1186/s12940-019-0459-5) contains supplementary material, which is available to authorized users.

## Background

Exposure to phenols and parabens has been linked to various adverse health effects, including ovarian toxicity, cancer, and adverse neurodevelopmental outcomes [1–4]. Prenatal exposure to these chemicals, in particular, may have a long lasting effect on fetal health into adulthood. For example, prenatal exposure to phenols and parabens has been linked to adverse birth outcomes [5, 6], respiratory health effects in children [7], and cardiometabolic risk [8]. The exact mechanisms at play are still not fully understood; however, endocrine disruption is hypothesized to be one of the main toxicity pathways [3, 9–11].

Reproductive and thyroid hormones play an essential role in the maintenance of pregnancy and the development of the fetus [12–16], therefore pregnancy is a vulnerable window for endocrine disruption due to the varying levels of hormones involved in the growing organism [17]. Endocrine disrupting chemicals could act through several pathways, including hormone synthesis, regulation, transport and metabolism, and/or interference with receptors. Phenols and parabens have estrogenic and androgenic properties [1, 18–20], but few human studies have looked into the effect of these chemicals on maternal hormones during pregnancy. Most existing studies in this area use smaller study populations or only examined a single time point in pregnancy, which do not capture the changing hormone levels and high variability of phenols and paraben exposure during pregnancy. Furthermore, no or few studies explored the associations between these chemicals and maternal testosterone, corticotropin-releasing hormone (CRH), sex hormone-binding globulin (SHBG) and estriol, all of which play essential roles in maintaining healthy pregnancies.

Given the growing evidence of the endocrine disrupting effects of phenols and parabens [18, 21–25], our aim was to study the relationships between phenols and parabens on reproductive and thyroid hormones in our ongoing cohort of pregnant women in Puerto Rico. The study follows the women over multiple time points during pregnancy, providing more power than previous studies, and allows for the identification of potential windows of susceptibility. We previously reported early preliminary results on associations between select phenols and parabens with hormones in this Puerto Rican cohort [26]. This manuscript is an update of our previous results that utilizes a much larger sample size, includes additional hormones (estriol, testosterone, total triiodothyronine, and total thyroxine), as well as additional exposure biomarkers yet to be studied in detail (ethylparaben, BPS, BPF and triclocarban). Due to the lack of human health data, this study was exploratory in nature, with the exception of BPA, triclosan, methylparaben and propylparaben. We hypothesized a decrease in serum thyroid hormone levels in association with triclosan, methyl- and propyl-paraben, and an increase in serum thyroid hormones with BPA concentrations.

## Methods

### Study participants

Participants for the present study were from an ongoing prospective cohort of pregnant women in Puerto Rico, named the Puerto Rico Testsite for Exploring Contamination Threats (PROTECT) cohort. Details on the recruitment and inclusion criteria have been described previously [27, 28]. The study participants included in the present analysis were recruited from 2012 to 2017 at 14 ± 2 weeks gestation from two hospitals and five affiliated prenatal clinics in Northern Puerto Rico. They were aged between 18 and 40 years. The exclusion criteria included women who lived outside the region, had multiple gestations, used oral contraceptives within 3 months prior to getting pregnant, got pregnant using in vitro fertilization, or had known medical health conditions (diabetes, hypertension, etc.). Three visits were conducted with the study participants to coincide with periods of rapid fetal growth and routine clinical visits (Visit 1: 16–20; Visit 2: 20–24; Visit 3: 24–28 gestation weeks). Demographic information was collected via questionnaires at the initial study visit. Spot urine samples were collected at the three study visits, whereas blood samples were collected during the first and third visits.

The present analysis includes 602 women recruited into the study (of the total 1311 women enrolled in the cohort to date) for whom both total phenol and paraben concentrations and hormone measurements from at least one study visit were available. This study was approved by the research and ethics committees of the University Of Michigan School Of Public Health, University of Puerto Rico, Northeastern University, and the University of Georgia. All study participants provided full informed consent prior to participation. The involvement of the Centers for Disease Control and Prevention (CDC) laboratory did not constitute engagement in human subjects research.

### Quantification of urinary biomarkers

After collection, spot urine samples were divided into aliquots and frozen at -80 °C until they were shipped overnight with dry ice to the CDC for analysis. Urine samples were analyzed for seven phenols (2,4-dichlorophenol, 2,5-dichlorophenol, BPA, BPS, BPF, benzophenone-3, triclosan), triclocarban, and four parabens (ethylparaben, methylparaben, butylparaben, propylparaben) using online solid phase extraction-high-performance liquid chromatography-isotope dilution tandem mass spectrometry [29–31]. Biomarker concentrations below the limit of detection (LOD) were assigned a value of the LOD divided by √2 [32]. The LODs were as follows: 0.1 μg/L (2,4-dichlorphenol, 2,5-dichlorophenol, BPS, triclocarban, butylparaben, propylparaben); 0.2 μg/L (BPA, BPF); 0.4 μg/L (benzophenone-3); 1 μg/L (methylparaben, ethylparaben); and 1.7 μg/L (triclosan). Urinary dilution was accounted for by using urinary specific gravity (SG), and was measured using a digital handheld refractometer (AtagoCo., Ltd., Tokyo, Japan). For preliminary data analysis, urinary biomarker concentrations were corrected for SG using the following formula:

$$ {\mathrm{P}}_{\mathrm{C}}=\mathrm{M}\left[\left({\mathrm{SG}}_{\mathrm{m}}-1\right)/\left({\mathrm{SG}}_{\mathrm{i}}-1\right)\right] $$

where P_c_ is the SG-corrected concentration (μg/L), M is the measured concentration, SG_m_ is the study population median urinary specific gravity (1.0196), and SG_i_ is the individual’s urinary specific gravity. The sample size for BPF, BPS, triclocarban and ethylparaben was smaller than the rest of the biomarkers because they were only quantified in a later sub-sample of the cohort.

### Hormone measurement

Serum samples were collected during visits 1 and 3. Volume limitations resulted in differences in the number of samples analyzed by hormone. All hormone analyses were conducted at the Central Ligand Assay Satellite Services (CLASS) laboratory, Department of Epidemiology, School of Public Health, University of Michigan. Progesterone, SHBG, testosterone, total triiodothyronine (T3), total thyroxine (T4), free thyroxine (FT4), and thyroid-stimulating hormone (TSH) were measured in serum using a chemiluminescence immunoassay (ADVIA Centaur® CP Immunoassay System, Seimens Healthineers). Estriol and CRH were measured in serum using an enzyme immunoassay (Estriol ELISA Kit, ALPCO; CRH ELISA Kit, LifeSpan BioSciences). In addition to measured hormones, the ratio of progesterone to estriol (Prog/Estriol Ratio), and the ratio of T3 and T4 (T3/T4 ratio) were calculated for the purposes of this analysis. Hormone ratios may be a better indicator of adverse pregnancy outcomes (such as preterm birth) than the individual hormones alone [33–35]. Two samples had a TSH level below the LOD. Because this result was not biologically plausible, these two values were dropped from the analyses.

### Statistical analyses

Distributions of key demographic characteristics were calculated. All urinary exposure biomarkers, and the serum hormones progesterone, estriol, CRH, TSH and progesterone/estriol ratio were positively-skewed, and were natural log-transformed. The distributions of SHBG, FT4, T3, T4 and T3/T4 ratio approximated normality and remained untransformed in all analyses. Geometric means and standard deviations were calculated for all SG-corrected exposure biomarkers, hormones, and the ratios of progesterone/estriol and T3/T4. We examined urinary exposure biomarkers concentrations and serum hormone levels by study visit, and calculated Spearman correlations between unlogged average SG-corrected exposure biomarkers. To assess differences in exposure biomarkers and hormones across study visits, we ran Linear Mixed Models (LMM) with a subject-specific random intercept regressing the biomarker or hormone against the study visit. Specific gravity was used as a covariate in the model instead of using the SG-corrected biomarker concentrations. The selection of a random intercept and slope was determined using BIC values. BPF and ethylparaben were detected in less than 50% of the samples. Therefore, we transformed BPF and ethylparaben into dichotomous variables, where 0 represented concentrations below the LOD, and 1 represented detectable concentrations. These categorical BPF and ethylparaben variables were used in all of the following regression analyses.

In our repeated measures analysis, we regressed one hormone or hormone ratio on one urinary biomarker using LMM, with a subject-specific random intercept for each model to account for intra-individual correlation of serial hormone measurements collected over the two study visits. The urinary biomarker concentrations at the two visits were treated as time-varying variables in the LMM models. Crude models included specific gravity and study visit as covariates. Potential confounders were selected a priori from the existing literature, and included as covariates if they were found to change the main effect estimate by > 10%. Final models were adjusted for specific gravity, study visit, body mass index (BMI) at the first study visit, maternal age, the number of hours of second-hand smoking exposure per day, and a socio-economic variable. All covariates, except for maternal age and specific gravity, were categorical. The socio-economic variable used in the model differed by the hormone regressed. Maternal education was a strong confounder for models regressing progesterone, estriol, and progesterone/estriol ratio against urinary biomarkers concentrations, and was used as the socio-economic index for those models. All other models used insurance type as the socio-economic status index. The selection of the socio-economic variable was based on the percent change in the main effect estimate, and the *p* value of the socio-economic variable in final models.

To assess windows of vulnerability, we ran two more analyses. First, we ran the same LMMs regressing hormones and urinary biomarkers concentrations with an interaction term between the urinary biomarker and the study visit. Second, we ran multiple linear regressions (MLR) stratified by study visit of sample collection. The MLR models were adjusted for the same covariates as those in the LMMs.

To increase interpretability of our results, we transformed regression coefficients to percent changes (and associated 95% confidence intervals, CIs) in hormone concentration in relation to the interquartile range (IQR) increase in urinary biomarker concentrations. Beta coefficients from models with categorical biomarkers (BPF and ethylparaben) were transformed to percent changes (and associated 95% confidence intervals) in hormone concentration at detectable vs non-detectable biomarker concentrations. The alpha level was set at 0.05. All statistical analyses were conducted in R Version 3.4.2.

As a sensitivity analysis, all models were re-run using specific gravity as a covariate in combination with exposure biomarkers corrected for specific gravity as was described by O’Brien et al. [36]. We observed no differences in our results, and therefore, retained our original models using un-corrected exposure biomarkers with specific gravity included as a covariate.

## Results

The 602 study participants had a mean age of 26.4 and approximately 60% had BMI levels below 30 kg/m^2^ (Table 1). Although the majority of women reported never smoking (75%), 4% reported currently smoking, and 7% reported exposure to second-hand smoking for more than an hour per day. Six percent reported consuming alcohol in the last few months. A quarter of the study participants reported a household income of less than $10,000, and only 11% reported a household income >$50,000. A quarter of the participants did not report their incomes. As compared to the overall PROTECT cohort, the study participants included in the present analysis had higher rates of smoking, and had overall lower household income and education levels.

**Table 1:** Summary demographics and differences between the PROTECT study participants included in present analysis versus participants not included because of missing urine and/or serum samples

Total N	Included	Not Included	p
	602	709	
Age (mean [SD])	26.51 (5.66)	26.94 (5.34)	0.25
BMI in kg/m^2^ (%)			
< 25	245 (40.7)	192 (27.1)	0.99
25–30	114 (18.9)	87 (12.3)	
> 30	73 (12.1)	56 (7.9)	
Missing	170 (28.2)	374 (52.8)	
Current Smoker (%)			
Never	440 (73.1)	323 (45.6)	0.03
Ever	63 (10.5)	57 (8.0)	
Current	23 (3.8)	6 (0.8)	
Missing	76 (12.6)	323 (45.6)	
Exposure to Second-Hand Smoking per Day (%)			
Up to half an hour	443 (73.6)	338 (47.7)	0.16
Up to an hour	25 (4.2)	19 (2.7)	
More than an hour	41 (6.8)	18 (2.5)	
Missing	93 (15.4)	334 (47.1)	
Alcohol Consumption (%)			
No	273 (45.3)	190 (26.8)	0.61
Before pregnancy	215 (35.7)	170 (24.0)	
Yes within the last few months	36 (6.0)	24 (3.4)	
Missing	78 (13.0)	325 (45.8)	
Household Income in U.S. $ (%)			
< 10,000	152 (25.2)	82 (11.6)	0.03
10,000 - 30,000	132 (21.9)	114 (16.1)	
30,000 - 50,000	101 (16.8)	83 (11.7)	
> 50,000	64 (10.6)	59 (8.3)	
Missing	153 (25.4)	371 (52.3)	
Maternal Education (%)			
< High School	123 (20.4)	64 (9.0)	0.02
Some college	194 (32.2)	137 (19.3)	
College graduate	210 (34.9)	182 (25.7)	
Missing	75 (12.5)	326 (46.0)	
Insurance Type (%)			
Public (Mi Salud)	318 (52.8)	340 (48.0)	0.001
Private	222 (36.9)	153 (21.6)	
Missing	62 (10.3)	216 (30.5)	

The exposure biomarkers included in this analysis were highly detected in the study population, with the exception of ethylparaben and BPF (Table 2). BPF was detected in between 50 and 60% of the study sample; ethylparaben was detected in between 42 and 54% of the sample, depending on study visit. Concentrations of urinary biomarkers remained relatively consistent across the two study visits, with the exception of a decrease in BPA (*p* < 0.001) and butylparaben (*p* = 0.04). There was an increase in most hormones across the two study visits, particularly progesterone, estriol, SHBG and CRH. T4 levels remained consistent from 16 to 20 and 24–28 weeks gestation.

**Table 2:** Distribution of SG-corrected urinary biomarker concentrations and hormones and differences by study visit of sample collection in pregnancy

Biomarkers^a^	16–20 weeks (*N* = 389)						24–28 weeks (*N* = 262)						*p*-value
	GM (GSD)	% < LOD	25%	50%	75%	95%	GM (GSD)	% < LOD	25%	50%	75%	95%	
2,4-DCP	1.17 (3.24)	0.5	0.52	0.93	2.0	10.7	1.13 (9.8)	2.3	0.46	0.86	2.19	12.9	0.65
2,5-DCP	14.03 (5.14)	0.3	4.57	10.4	30.2	432.6	13.63 (360.3)	0	4.63	9.61	26.53	429.6	0.70
BPA	2.31 (2.25)	0.3	1.33	2.14	3.36	9.56	1.88 (2.4)	0.8	1.14	1.83	3.0	6.18	< 0.001*
BPS^i^	0.54 (3.15)	3.4	0.23	0.50	1.07	4.01	0.54 (5.2)	8.6	0.23	0.47	1.06	4.23	0.95
BPF^i^	0.35 (3.18)	51.9	<LOD	0.25	0.56	2.88	0.31 (2.4)	59.9	<LOD	0.24	0.46	2.09	0.22
BP-3	38.34 (6.49)	0.5	10.6	22.4	110.8	1547	44.27 (2500.4)	0.8	11.9	25.3	160.7	1913.5	0.69
TCS	21.78 (8.72)	11.1	3.70	13.1	146.8	877.4	25.03 (327.4)	6.1	4.73	17.9	118.6	960.2	0.64
TCC^i^	4.34 (10.27)	5.8	0.70	3.36	33.6	157.7	4.86 (56.2)	5.6	0.78	4.78	32.76	168.6	0.46
EPB^i^	3.42 (7.73)	42.4	<LOD	1.66	15.4	177.5	2.55 (62.1)	54	<LOD	<LOD	7.6	76.1	0.12
MPB	80.72 (5.06)	0.3	25.08	116.5	274.8	846.1	92.5 (359)	0.8	30.2	111	314.5	1054.9	0.18
BPB	0.55 (8.12)	23.8	0.10	0.25	2.17	39.1	0.42 (12.2)	33.5	0.10	0.2	0.91	32.6	0.04*
PPB	17.51 (7.19)	0	3.59	21.1	80.5	262.4	17.61 (111.4)	0.4	4.0	25.17	83.65	253.8	0.99
Hormones	16–20 weeks (*N* = 483)						24–28 weeks (N = 389)						*p*-value
	GM (GSD)	% < LOD	25%	50%	75%	95%	GM (GSD)	% < LOD	25%	50%	75%	95%	
Progesterone^b^	49.63 (1.49)	0	37.2	48.5	61.6	98.2	95.94 (1.65)	0	70.9	90.9	129.2	222.7	< 0.001*
Estriol^b^	18.85 (1.76)	0	13.2	17.6	27.5	50.5	44.63 (1.59)	0	33.8	44.9	57.5	97.3	< 0.001*
SHBG ^c^	575.22 (1.4)	0	482.4	588.6	703.8	907.9	670.19 (1.35)	0	536.3	672.9	832.6	1097.7	< 0.001*
Prog/Estriol	2.64 (1.69)	–	1.97	2.66	3.70	5.83	2.15 (1.62)	–	1.58	2.12	2.86	4.48	< 0.001*
CRH^d^	76.67 (1.70)	0	54.8	80.6	111.8	171.9	78.01 (1.76)	0	55.2	82.8	114.3	178.1	< 0.001*
Testosterone^f^	50.55 (1.81)	1.8	37.5	51.5	73.5	124.4	60.22 (1.75)	0.9	45.1	60.7	88.1	131.6	< 0.001*
TSH^e^	1.29 (2.14)	0	0.93	1.38	2.06	3.28	1.45 (1.79)	0	1.08	1.51	2.05	3.64	0.04*
FT4^f^	1.10 (1.13)	0	1.02	1.10	1.19	1.35	1.06 (1.13)	0	0.98	1.06	1.15	1.29	< 0.001*
T3^b^	1.94 (1.22)	0	1.71	1.97	2.22	2.59	1.95 (1.21)	0	1.72	1.99	2.24	2.67	0.03*
T4 ^g^	11.90 (1.20)	0	10.7	11.95	13.3	15.7	11.71 (1.20)	0	10.5	11.7	13.2	15.5	0.27
T3/T4	0.16 (1.21)	–	0.14	0.16	0.19	0.23	0.17 (1.22)	–	0.15	0.17	0.19	0.23	< 0.001*

*GM* Geometric mean, *GSD* Geometric standard deviation

2,4-DCP: 2,4-dichlorophenol; 2,5-DCP: 2,5-dichlorophenol; *BP-3* Benzophenone, *TCS* Triclosan, *TCC* Triclocarban, *EPB* ethylparaben, *MPB* Methylparaben, *BPB* Butylparaben, *PPB* Propylparaben

Range of gestational weeks: 16–20 weeks: 16–20 weeks gestation, 24–28 weeks: 24–28 weeks gestation

^*^Significant difference (*p* < 0.05) in urinary biomarker or hormone compared to reference (16–20 weeks) using linear mixed models with a random intercept

^a^Units: μg/L. ^b^ Units: ng/mL. ^c^ Units: nmol/L. ^d^ Units: pg/mL. ^e^ Units: uIU/mL. ^f^ Units: ng/dL. ^g^ Units: μg/dL. ^I^ BPS, BPF, TCC and EPB had the lowest sample sizes because they were added to the biomarker assay at mid-study. At 16–20 weeks, these four urinary biomarkers had *N* = 295. At 24–28 weeks, these four urinary biomarkers had *N* = 198

**Fig. 1:**
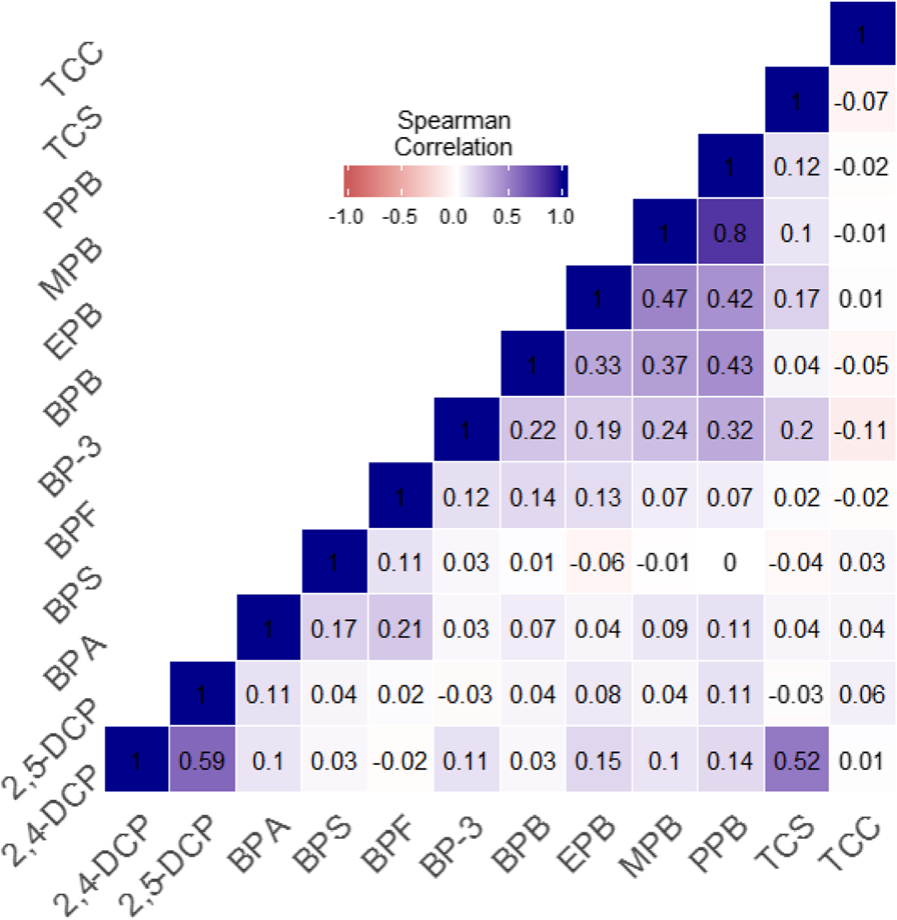
Heat map of Spearman correlations between unlogged urinary triclocarban, phenols and parabens. Biomarkers concentrations were adjusted for urinary dilution. 2,4-DCP: 2,4-dichlorophenol; 2,5-DCP: 2,5-dichlorophenol; BP-3: Benzophenone; TCS: Triclosan; TCC: Triclocarban; EPB: ethylparaben; MPB: Methylparaben; BPB: Butylparaben; PPB: Propylparaben

Methylparaben and propylparaben were strongly correlated [Spearman correlation of 0.8 (*p* < 0.001)] (Fig. 1). Ethylparaben and butylparaben showed moderate correlation with methylparaben and propylparaben with Spearman correlations between 0.33–0.47 (*p* values < 0.001). 2,4-Dichlorophenol and 2,5-dichlorophenol showed a fairly strong correlation (Spearman *r* = 0.6, *p* < 0.001). Triclosan was moderately correlated with 2,4-dichlorophenol (Spearman *r* = 0.5, *p* < 0.001), but not with 2,5-dichlorophenol (Spearman *r* = − 0.03). BPA, BPS and BPF showed low correlation (Spearman r = 0.11–0.21, *p* < 0.001).

Results from LMMs and MLRs are described in detail below by biomarker (Tables 3, Additional file 1: Table S1 and S2, and Additional file 2). There were few differences between most adjusted and unadjusted models, with the exception of associations with CRH. MPB and PPB were associated with CRH in our unadjusted models, but in the adjusted models, these associations disappeared, and CRH was associated with BPS and TCS. A further analysis of CRH concentrations across the covariate levels did not reveal any large differences to report.

**Table 3:** Results of the adjusted LMMs regressing hormones versus exposure biomarkers

		CRH	SHBG	Testosterone	Progesterone	Estriol	Progesterone/Estriol Ratio
2,4-DCP	% Δ/IQR	5.30 (− 2.81, 14.08)	2.06 (− 1.49, 5.61)	3.26 (− 3.01, 9.94)	1.60 (− 3.42, 6.87)	− 1.93 (− 7.54, 4.01)	3.58 (− 1.85, 10.16)
	p	0.21	0.26	0.32	0.54	0.52	0.24
2,5-DCP	% Δ/IQR	3.89 (− 3.29, 11.61)	0.96 (− 2.25, 4.17)	1.75 (− 3.85, 7.69)^a^	− 0.46 (− 4.79, 4.07)	− 2.21 (− 7.12, 2.96)	1.35 (− 3.46, 6.82)
	p	0.30	0.56	0.55	0.84	0.40	0.61
BPA	% Δ/IQR	3.68 (−4.21, 12.22)	− 0.22 (− 3.60, 3.15)	− 4.19 (− 9.64, 1.59)^a^	− 3.50 (− 8.16, 1.39)	− 2.18 (− 7.78, 3.78)	−1.55 (− 6.18, 5.01)
	p	0.37	0.90	0.15	0.16	0.47	0.60
BPF^b^	% Δ/IQR	3.90 (−9.72, 19.57)	− 2.97 (− 8.04, 2.11)	0.33 (−8.89, 10.49)	−1.33 (− 23.9, 13.78)	3.84 (− 6.40, 15.21)	− 4.65 (− 13.44, 5.05)
	p	0.60	0.26	0.95	0.76	0.48	0.34
BPS	% Δ/IQR	−11.35 (− 18.71, − 3.33)	− 0.56 (− 4.37, 3.25)	2.54 (− 3.5, 8.97)	− 4.38 (− 9.49, 1.02)	− 2.05 (− 8.16, 4.47)	− 2.96 (− 7.85, 3.85)
	p	0.008**	0.77	0.42	0.11	0.53	0.34
BP-3	% Δ/IQR	− 0.04 (− 7.96, 8.57)	1.10 (− 2.61, 4.82)	− 0.51 (− 6.8, 6.21)	0.46 (− 4.62, 5.81)	− 0.91 (− 6.66, 5.18)	1.81 (− 3.60, 8.42)
	p	0.99	0.56	0.88	0.86	0.76	0.56
TCC	% Δ/IQR	−3.69 (− 14.5, 8.50)	− 4.54 (− 10.03, 0.94)	5.18 (− 3.4, 14.51)	−3.22 (− 10.13, 4.21)	0.36 (− 7.93, 9.39)	−3.75 (− 8.64, 7.7)
	p	0.54	0.11	0.25	0.39	0.94	0.39
TCS	% Δ/IQR	9.20 (− 0.97, 20.42)	2.81 (− 1.46, 7.08)	7.13 (− 0.60, 15.5)	2.84 (−3.2, 9.25)^a^	4.16 (− 3.07, 11.93)^a^	0.31 (− 5.8, 8.4)
	p	0.08*	0.20	0.07*	0.37	0.27	0.93
EPB^b^	% Δ/IQR	1.52 (−11.55, 16.52)	−1.93 (−8.14, 4.29)	5.11 (− 4.64, 15.86) ^a^	−2.41 (− 10.62, 6.56)	−1.92 (− 11.4, 8.58)	− 0.77 (− 10.22, 9.67)
	p	0.83	0.55	0.32	0.59	0.71	0.88
BPB	% Δ/IQR	−1.86 (− 10.64, 7.8)	−5.27 (−9.4, − 1.14)	− 6.77 (− 13.3, 0.29)	−3.65 (− 9.11, 2.14)	−5.18 (− 11.45, 1.52)	1.96 (− 4.9, 8.52)
	p	0.70	0.01**	0.06*	0.21	0.13	0.58
MPB	% Δ/IQR	5.88 (− 3.0, 15.59)	−3.53 (− 7.37, 0.31)	−4.41 (− 10.68, 2.3)	0.03 (−5.29, 5.64)	−2.50 (− 8.6, 4.01)^a^	2.64 (− 3.06, 9.74)
	p	0.20	0.07*	0.19	0.99	0.44	0.43
PPB	% Δ/IQR	4.82 (−4.48, 15.02)	−3.74 (−7.76, 0.27)	− 3.54 (− 10.14, 3.54)	2.35 (− 3.55, 8.6)	−0.63 (− 7.36, 6.58)^a^	3.65 (− 2.36, 11.66)
	p	0.32	0.07*	0.32	0.44	0.86	0.31
		TSH	FT4	T3	T4	T3/T4 ratio	
2,4-DCP	% Δ/IQR	4.80 (−2.58, 12.74)	0.21 (−1.19, 1.60)	− 1.58 (−3.58, 0.42)	−0.79 (− 2.71, 1.13)	−1.16 (− 4.86, 1.33)	
	p	0.21	0.77	0.12	0.42	0.31	
2,5-DCP	% Δ/IQR	4.63 (− 2.08, 11.79)	0.82 (−0.43, 2.07)	−0.55 (− 2.36, 1.26)	0.51 (−1.22, 2.24)	−1.38 (−4.64, 1.06)	
	p	0.18	0.2	0.56	0.57	0.18	
BPA	% Δ/IQR	−0.28 (−6.99, 6.91)	0.00 (− 1.36, 1.36)	2.10 (0.22, 3.99)	0.69 (−1.13, 2.51)	1.46 (−2.23, 3.52)	
	p	0.94	1	0.03**	0.46	0.19	
BPF^b^	% Δ/IQR	7.29 (−4.59, 20.64)	2.76 (0.29, 5.22) ^a^	−1.22 (− 4.34, 1.90)	1.84 (−1.37, 5.04)	−2.50 (−6.47, 1.47)	
	p	0.24	0.03**	0.45	0.26	0.22	
BPS	% Δ/IQR	−1.01 (−8.12, 6.66)	−0.07 (− 1.6, 1.46)	0.14 (− 1.89, 2.18)	0.04 (− 1.97, 2.05)	0.58 (− 2.47, 2.9)	
	p	0.79	0.93	0.89	0.97	0.64	
BP-3	% Δ/IQR	−5.89 (−12.87, 1.65)	− 0.40 (− 1.85, 1.04)	−1.47 (−3.56, 0.63)	−1.37 (− 3.35, 0.62)	−0.21 (− 5.33, 1.26)	
	p	0.13	0.59	0.17	0.18	0.86	
TCC	% Δ/IQR	−10.12 (− 19.47, 0.32)	−0.61 (−2.76, 1.55)	4.08 (1.18, 6.98)	− 0.65 (− 3.53, 2.23)	4.67 (− 1.37, 6.65)	
	p	0.06*	0.58	0.007**	0.66	0.01**	
TCS	% Δ/IQR	0.57 (−7.74, 9.63)	−0.74 (−2.45, 0.96)	− 1.97 (−4.36, 0.41)	−1.60 (−3.9, 0.69)	−0.15 (− 2.69, 4.55)	
	p	0.9	0.39	0.11	0.17	0.91	
EPB^b^	% Δ/IQR	−6.78 (−17.6, 5.46)	−0.45 (−2.9, 2.0)	−0.76 (−4.10, 2.58)	−0.03 (−3.29, 3.24)	− 1.68 (−5.66, 2.30)	
	p	0.27	0.72	0.66	0.99	0.41	
BPB	% Δ/IQR	−4.88 (− 12.62, 3.54)	1.10 (−0.54, 2.74)	0.70 (− 1.61, 3.02)	1.74 (−0.49, 3.96)	− 1.56 (− 7.01, − 0.26)	
	p	0.25	0.19	0.55	0.13	0.24	
MPB	% Δ/IQR	−6.92 (−13.91, 0.64)^a^	0.77 (−0.76, 2.29)	−0.39 (− 2.53, 1.76)	1.02 (− 1.04, 3.09)	−1.78 (−4.64, 1.53)	
	p	0.07*	0.33	0.73	0.33	0.15	
PPB	% Δ/IQR	−6.29 (− 13.6, 1.64)	0.81 (−0.8, 2.42)	0.21 (−2.02, 2.45)	0.65 (− 1.52, 2.81)	− 0.67 (− 3.89, 2.63)	
	p	0.12	0.32	0.85	0.56	0.6	

2,4-DCP: 2,4-dichlorophenol; 2,5-DCP: 2,5-dichlorophenol; BP-3: Benzophenone; TCS: Triclosan; TCC: Triclocarban; EPB: ethylparaben; MPB: Methylparaben; BPB: Butylparaben; PPB: Propylparaben

Results converted to % change in hormone per IQR change in biomarker concentration

* represents a p value below 0.10, and **represents a p value below 0.05; a Significant interaction (p < 0.05) between urinary biomarker*visit; b Dichotomous variable

Models adjusted for specific gravity, study visit, body mass index (BMI) at the first study visit, maternal age, the number of hours of second-hand smoking exposure per day, and a socio-economic variable

There were no associations between 2,4-dichlorophenol and 2,5-dichlorophenol with hormones in LMMs. An IQR increase in 2,4-dichlorophenol was associated with a 10% increase in CRH at 24–28 weeks [9.66% change in hormone per IQR change in the biomarker/ percent change in hormone at detectable biomarker concentrations (%Δ); 95% CI: 0.67, 19.45], and a suggestive 2% decrease in T3 at 16–20 weeks (%Δ -2.22 95% CI -4.55, 0.10).

Associations across the bisphenols differed, and BPS had the strongest associations in LMM models. BPS was associated with an 11% decrease in CRH (%Δ -11.35; 95% CI: -18.71, − 3.33), and this association was stronger at 16–20 weeks gestation. At this time point, BPS was additionally associated with a 12% decrease in TSH (%Δ -11.93; 95% CI: -22.49, 0.07). BPF was associated with a 3% increase in FT4 (%Δ 2.76; 95% CI: 0.29, 5.22), and this association was also stronger at 16–20 weeks. BPA, on the other hand, had stronger associations at 24–28 weeks gestation. BPA was associated with a 17% decrease in testosterone, and 2–4% increases in FT4 and T3 at 24–28 weeks [(%Δ -17.37; 95% CI: -26.7, − 6.87); (%Δ 2.38; 95% CI: 0.04, 4.72); (%Δ4.33, 95% CI: 0.11, 8.55), respectively]. The increase in FT4 and T3 in relation to BPA was in line with our a priori hypothesis Benzophenone-3 was not significantly associated with any hormones.

Triclocarban was associated with a number of thyroid hormones and SHBG. An IQR increase in triclocarban is associated with a 4% increase in T3 (%Δ 4.08; 95% CI: 1.18, 6.98), a 5% increase in the T3/T4 ratio (%Δ 4.67; 95% CI: 1.24, 10.10), a suggestive 10% decrease in TSH (%Δ -10.12; 95% CI: -19.47, 0.32), and a 10% decrease in SHBG at 24–28 weeks (%Δ -9.71; 95% CI: -19.1, − 0.27).

Triclosan was associated with an increase in a number of reproductive hormones, however most were only suggestive with *p* values between 0.05 and 0.10. This includes a 9% increase in CRH (%Δ 9.20; 95% CI: -0.97, 20.42), a 7% increase in testosterone (%Δ 7.13; 95% CI: -0.60, 15.5), and 10–13% increases in progesterone and estriol at 24–28 weeks [(%Δ 9.72, 95% CI: -1.27, 21.9); (%Δ 13.2; 95% CI: 2.34, 25.2), respectively]. In addition, triclosan was associated with a 5.8% decrease in T3 at 24–28 weeks; this finding was in line with our a priori hypothesis.

IQR increases in butylparaben, methylparaben and propylparaben were associated with a decrease in SHBG [(%Δ -5.27; 95% CI:-9.40, − 1.14); (%Δ -3.53; 95% CI: -7.37, 0.31); (%Δ -3.74; 95% CI: -7.76, 0.27), respectively]. Methylparaben was also associated with decreases in reproductive hormones, including an 8% decrease in estriol, a suggestive 3% increase in the progesterone/estriol ratio, and a suggestive decrease in testosterone at 16–20 weeks [(%Δ -7.76; 95% CI: -15.4, 0.61); (%Δ 3.14; 95% CI: -2.95, 9.61); (%Δ -6.77; 95% CI: -13.13, 0.29), respectively]. Conversely, an IQR increase in propylparaben was associated with a 9–10% *increase* in progesterone and estriol at 24–28 weeks [(%Δ 9.67; 95% CI: -1.30, 21.85); (%Δ 8.92; 95% CI: -1.56, 20.52)]. Interaction terms between study visit*methylparaben and propylparaben had *p* values < 0.05 in models regressed against estriol. We expected to see a decrease in thyroid hormones in relation to methyl- and propyl- paraben, but only observed a decrease in TSH in association with methylparaben, particularly at 16–20 weeks (%Δ -11.69; 95% CI: -21.97, − 0.06). The decrease in TSH could indicate an increase in circulating thyroid hormones, in contrast to our hypothesis.

## Discussion

Associations differed by exposure biomarker and hormone, and there was little consistency within chemical classes with the exception of some parabens. There was evidence of a decrease up to 6% in T3 in association with 2,4-dichlorophenol, BPA and triclosan, whereas triclocarban was associated with a 4% increase in T3. In the case of bisphenols, BPS was more strongly related to decreases in hormones at 16–20 weeks, and BPA had stronger negative relationships at 24–28 weeks. Triclosan was associated with general increases in reproductive hormones of approximately 10%, and triclocarban was associated with 5–10% changes in thyroid hormones. Parabens were associated with a decreased level of SHBG.

While there may be structural similarities between BPA, BPS and BPF, the structural variations may be sufficient to alter receptor-binding affinities across the bisphenols [37]; therefore, the biological effects may vary among the bisphenols. To this, we found that the earlier time point (16–20 weeks gestation) may be a more vulnerable time of exposure to BPS and BPF, in contrast to the stronger relationships observed at the 24–28 weeks with respect to BPA. Our results were somewhat consistent with results from previous studies. BPA has been suspected to interfere with thyroid hormones, as evidenced by several epidemiological studies. We observed an increase in FT4 and T3, which was consistent with two previous studies our group conducted in a preliminary analysis in the PROTECT cohort, and a cohort of pregnant women in Boston, MA with four repeated measures during pregnancy [38, 39]. Two cross-sectional studies in the United States (*N* = 249 and 476 women) also looked at the association between maternal BPA and thyroid hormones during gestation [40, 41]. The only significant association reported was between maternal urinary BPA and a decrease in T4 [40], which we did not observe in the present study. A decrease in T4 could be indicative of an increase in FT4, in the case of thyroxine becoming less bound to thyroxine-binding globulin, however, the associations between BPA and T4 in the current study had *p* values ranging from 0.51–0.93. Furthermore, we did not observe a relationship between BPA and TSH that was reported in the Boston cohort study [42], and among adults from the Korean National Environmental Health Survey [43].

One of the strongest associations we observed was the 17% decrease in testosterone in relation to BPA. This is the first study that explores this association in pregnant women, and there is little correlation between maternal and fetal testosterone levels [44]. However, a decrease in testosterone was identified in an in vitro study on TM3 murine Leydig with BPA exposure [45], in the F2 generation after in-utero BPA exposure in mice [46], and in-utero BPA concentrations in young boys aged 8–14 [47]. These associations provide further evidence in support of our finding. Although the role of maternal testosterone in gestation is still unclear, evidence points to androgens playing an essential role in myometrial relaxation, cervical ripening and initiating parturition [48]. Therefore, BPA, via reduced testosterone, could increase gestational age, which we previously observed in this cohort [49]. Additionally, maternal testosterone has a role in gender role behaviors [50], indicating that maternal testosterone may impact fetal development.

No human studies have previously investigated the associations between triclocarban, phenols and parabens on CRH during pregnancy; however, CRH plays an important role in gestation. Maternal CRH levels during pregnancy largely originate from gestational tissues [51]. Evidence suggests CRH inhibits immune rejection processes by killing activated T cells [52], plays an important role in determining time of parturition, and an increase in CRH has been associated with the onset of miscarriage and preeclampsia [53–57]. CRH receptor expression is regulated by estrogen, and CRH gene expression in the placenta is mediated by ER-α [58, 59]. Given the endocrine disrupting potential of bisphenols via estrogen receptors [60], associations between CRH and bisphenols (and potentially other phenols and parabens) could be important to consider in pregnancy studies. Animal and in vitro studies showed an increase in CRH with exposure to BPA and BPS, contrary to our results of an inverse relationship between CRH and BPS. BPA increased plasma concentrations of CRH in pregnant mice [61] and CRH levels in human placenta primary trophoblast cells [62]. The differences in our results could be in part due to the unique role CRH plays in human pregnancies, as compared to animals [63].

Triclosan was suggestively associated with select hormones, but none reached statistical significance, including an increase in testosterone, an increase in CRH at 16–20 weeks gestation, and a decrease in T3 at 24–28 weeks gestation. There was a similar decrease in T3 with increased urinary triclosan concentrations in the Boston cohort, albeit the associations were stronger earlier in pregnancy, in contrast to our stronger associations at the later visit in the current study [39]. While larger human studies with more statistical power may be needed, the decrease in T3 in association with triclosan is consistent with animal studies [64], including in pregnant rats [65] and pregnant mice [66, 67], perhaps due to triclosan’s structural similarities to thyroid hormones [64]. Animal studies also report a decrease in T4 with triclosan exposure, including rat and mice dams [65–75], but we did not find evidence of this in humans. Other population studies found no associations between triclosan and thyroid hormones [76–78], although there was evidence of vulnerable time points during gestation [76, 77]. Interestingly, a study in pregnant rats showed that the greatest accumulation of triclosan was in the placenta, indicating that pregnancy may be a sensitive time period for triclosan exposure [79]. Alternatively, maternal serum TSH and FT4 levels at > 28 weeks gestation (obtained from medical records) were negatively associated with urinary triclosan at 38 weeks gestation [80]. The differences in our results could be explained by the differences in the study population, exposure biomarker concentrations, and differences in the pregnancy time points examined.

No studies have looked at the effect of triclosan on maternal testosterone and CRH during pregnancy in humans. However, in contrast to our results, triclosan was found to reduce testosterone levels in male rats [81], and in pregnant rats [79]. An excess of maternal testosterone has been associated with restricted fetal growth [82], as well as an increased chance of developing Alzheimer disease [83] and anxiety like symptoms in the offspring.

Triclocarban was associated with thyroid hormone changes. We observed an increase in T3 and a decrease in TSH in association with triclocarban, which is in line with the negative feedback loop in maintaining thyroid hormone homeostasis. We also observed a decrease in SHBG. SHBG levels tend to rise with thyroid hormones, so this observed pattern was unexpected. This could be due to factors influencing the relationship between thyroid hormone and SHBG levels that have not been accounted for in the present study. Our previous Boston study also reported a negative association between triclocarban and TSH, but a negative association with T3. Triclocarban concentrations in this cohort were much higher than the exposure levels found in the Boston cohort. In fact, the triclocarban concentrations observed in PROTECT are 37 times larger than the concentration observed in NHANES women of reproductive age [84]. This difference in exposure levels may explain the differences in the associations observed.

All parabens were generally negatively associated with SHBG. In contrast to our current findings, our previous preliminary analysis in the PROTECT cohort showed that methylparaben was positively associated with SHBG [26]. However, the current study has a much larger sample size. Associations between parabens and some hormones appeared to be dependent on the timing of exposure. Associations between methylparaben and propylparaben and estriol changed direction from a negative association at 16–20 weeks to a positive association at 24–28 weeks gestation. We observed a similar change in direction in our preliminary analyses between methylparaben and propylparaben with estradiol [26]. Although not statistically significant, associations between methylparaben and propylparaben with progesterone followed a similar pattern to that of estriol. Given that the population urinary levels of methylparaben and propylparaben remained consistent between the two time points, the similar change of direction observed in associations with methylparaben and propylparaben in both of our previous analyses, and the significant interaction term between these parabens and visit in association with estriol, this lends confidence that these observations may not be occurring by chance and may be detected in future larger studies. The strong correlation between propyl- and methylparaben could indicate that their associations with estriol are being driven by only one of the parabens. However, given the differences in the associations between these two parabens and all hormones, there do seem to be unique relationships between the exposure and hormone levels. No previous studies have looked at the effect of parabens on estriol, SHBG or CRH; however, evidence suggests parabens have ER-β agonistic activity [85], and stimulate progesterone mRNA expression via ER-α signaling [86, 87]. This could suggest a potential mechanism by which reproductive hormone levels could be directly or indirectly altered in response to paraben exposure.

The present study also showed a general decrease in TSH in association with parabens, but only methylparaben reached a significant association with TSH. Additionally, methylparaben and propylparaben were associated with a decrease in the T3/T4 ratio, particularly at 24–28 weeks gestation. Results from our Boston cohort also showed a decrease in T3/T4 ratio, as well as T3, at median 26 weeks gestation [88]. In other research, human and animal studies reported a decrease in T4 and FT4 with paraben exposure in females [78, 89], and two small studies in men found no associations between parabens and thyroid hormones [90, 91]. The difference in the results is likely due to the different study populations; none of those studies looked specifically at prenatal exposure.

Our study had several limitations. We did not have data on the iodine status of the women; deficiency in this element could affect thyroid hormone function. However, iodine may act as mechanistic intermediate exposure between the exposure and thyroid hormone, and controlling for iodine status could lead to bias [92]. Furthermore, iodine had no effect on the associations between phenols and thyroid hormones in our previous study of NHANES data [78]. We also did not have data on thyroperoxidase antibodies nor human chorionic gonadotropin (hCG), which could potentially affect thyroid function as well [93, 94]. While data at two time points is a great improvement from the more common cross-sectional study design, the two time points may not be sufficient to understand the potential influence of these biomarkers on maternal hormones. The relatively high variation in urinary concentrations of the target biomarkers (particularly BPA) over time may also introduce potential bias stemming from random measurement error. Given the multiple comparisons conducted, there is a chance of Type I error, and caution must be used when interpreting our findings. Finally, although one of the strengths of the present study is our ability to investigate the relationships between these chemicals and hormone levels in a vulnerable population, our study population was based in a population in Puerto Rico of lower income who also had higher urinary concentrations of some of the exposure biomarkers; therefore, the results may not be fully generalizable to other populations.

Our study also had many strengths. Our robust sample size, and the collection of exposure biomarkers and hormone data at two time points during pregnancy helps account for the biomarkers’ short lifespan in the body, and the varying levels of hormones throughout pregnancy. The repeated measures allow for the control of intra-individual variability, and increases statistical power. We were also able to explore potential windows of susceptibility for these associations.

Additionally, we were able to compare our results from this analysis to our own analyses that employed similar statistical methods in two other data sets, namely LMMs to capture biomarkers at various time points and allow subject-specific intercepts. While there were many similarities in the results across the three analyses, the differences in results may point to the importance of outside factors that may not be captured in our models that alter the associations between these chemicals and endocrine disruption through interaction with the chemicals. These outside factors could include other endocrine-altering variables, such as exposure to other unaccounted for chemicals, maternal stress, genetic, epigenetic, or other differences. It is imperative that future studies look beyond the association between a single chemical and singe hormone, and explore potential interactions with chemical exposure.

## Conclusion

Our results provide suggestive human evidence for associations between select biomarkers with maternal thyroid and reproductive hormones during gestation. Of note, we report negative associations between parabens and SHBG, a negative association between BPS and CRH, and associations between triclocarban and triclosan with reproductive and thyroid hormones. Our stratified analyses show that some associations may be stronger at certain time points during pregnancy. Further studies in larger populations and with more repeated measures across pregnancy to will be useful to confirm our findings, and better understand if and how these hormone changes may affect downstream maternal and infant health outcomes.

## Additional file

Additional file 1: **Table S1.** Results of the adjusted MLRs regressing reproductive hormones versus exposure biomarkers by visit. **Table S2.** Results of the adjusted MLRs regressing thyroid hormones versus exposure biomarkers by visit. **Table S3.** Result comparison between the common exposure biomarkers and hormones. (DOCX 32 kb)

Additional file 2: Adjusted multiple linear regressions of hormones versus urinary concentrations of biomarkers stratified by study visit. Visit 1: 16-20 weeks; Visit 3: 24-28 weeks. EPB and BPF are categorical variables. * represents at least one marginal association between the urinary concentration and the hormone across the four time points. ** represents at least one significant association between the urinary biomarker concentration and the hormone across the four time points. BPF and EPB were dichotomous variables. 2,4-DCP: 2,4-dichlorophenol; 2,5-DCP: 2,5-dichlorophenol; BP-3: Benzophenone; TCS: Triclosan; TCC: Triclocarban; EPB: ethylparaben; MPB: Methylparaben; BPB: Butylparaben; PPB: Propylparaben (DOCX 664 kb)

## Abbreviations

BMI: Body mass index
BPA: Bisphenol-A
BPF: Bisphenol-F
BPS: Bishphenol-S
CDC: Centers for Disease Control and Prevention
CI: Confidence intervals
CRH: Corticotropin-releasing hormone
FT4: Free thyroxine
hCG: Human chorionic gonadotropin
LMM: Linear Mixed Models
LOD: Limit of detection
MLR: Multiple linear regression
NHANES: National Health and Nutrition Examination Survey
PROTECT: Puerto Rico Testsite for Exploring Contamination Threats
SG: Specific gravity
SHBG: Sex-hormone-binding globulin
T3: Total triiodothyronine
T4: Total thyroxine
TSH: Thyroid-stimulating hormone
