# Supplementary material for: A repeated measures study of phenol, paraben and Triclocarban urinary biomarkers and circulating maternal hormones during gestation in the Puerto Rico PROTECT cohort
Source: Environ Health. 2019 Apr 2;18:28. doi: 10.1186/s12940-019-0459-5 (PMC6444601; doi:10.1186/s12940-019-0459-5)
Supplement: Supplementary file 1 — Table S1. Results of the adjusted MLRs regressing reproductive hormones versus exposure biomarkers by visit. Table S2. Results of the adjusted MLRs regressing thyroid hormones versus exposure biomarkers by visit. Table S3. Result comparison between the common exposure biomarkers and hormones. (DOCX 32 kb) [file 12940_2019_459_MOESM1_ESM.docx]

# **Appendix**

# **Supplementary Tables for “A Repeated Measures Study of Phenol, Paraben and Triclocarban Urinary Biomarkers and Circulating Maternal Hormones during Gestation in the Puerto Rico PROTECT Cohort” by Aker et al, 2018**

Supplementary Table 1: Results of the adjusted MLRs regressing reproductive hormones versus exposure biomarkers by visit.

|  | | **16-20 weeks gestation** | | | | | |
| --- | --- | --- | --- | --- | --- | --- | --- |
|  |  | **CRH** | **SHBG** | **Testosterone** | **Progesterone** | **Estriol** | **Progesterone/Estriol Ratio** |
| **2,4-DCP** | % Δ/IQR | 2.93 (-6.84, 13.73) | 0.18 (-3.39, 3.74) | 3.66 (-3.47, 11.32) | -0.33 (-4.75, 4.29) | -3.54 (-9.78, 3.12) | 3.70 (-2.35, 11.15) |
|  | p | 0.57 | 0.92 | 0.32 | 0.92 | 0.29 | 0.89 |
| **2,5-DCP** | % Δ/IQR | 5.20 (-4.94, 16.43) | -0.16 (-3.79, 3.47) | 2.11 (-5.04, 9.8)^a^ | 0.45 (-4.05, 5.17) | -0.92 (-7.4, 6.01) | 1.48 (-5.03, 8.31) |
|  | p | 0.33 | 0.93 | 0.57 | 0.94 | 0.79 | 0.86 |
| **BPA** | % Δ/IQR | 6.35 (-4.08, 17.92) | 0.09 (-3.64, 3.81) | 2.77 (-4.58, 10.69)^a^ | -2.11 (-6.36, 2.34) | 0.17 (-6.4, 7.21) | -2.55 (-8.2, 4.6) |
|  | p | 0.24 | 0.96 | 0.47 | 0.45 | 0.96 | 0.38 |
| **BPF^b^** | % Δ/IQR | 10.11 (-11.4, 36.81) | -6.81 (-14.8, 1.16) | 3.71 (-8.85, 18.01) | -1.45 (-27.8, 36.5) | 7.50 (-7.52, 24.96) | -8.14 (-17.62, 2.43) |
|  | p | 0.39 | 0.1 | 0.58 | 0.97 | 0.35 | 0.79 |
| **BPS** | % Δ/IQR | -16.15 (-26.6, -4.2) | -2.37 (-7.36, 2.61) | 3.08 (-5.01, 11.86) | -4.51 (-9.77, 1.05) | -3.16 (-11.46, 5.92) | -1.48 (-8.62, 8.97) |
|  | p | 0.01** | 0.35 | 0.47 | 0.35 | 0.48 | 0.13 |
| **BP-3** | % Δ/IQR | -2.66 (-12.44, 8.2) | 1.25 (-2.53, 5.04) | 2.69 (-4.78, 10.75) | -0.60 (-5.27, 4.29) | -3.14 (-9.84, 4.06) | 2.22 (-4.1, 9.93) |
|  | p | 0.62 | 0.52 | 0.49 | 0.72 | 0.38 | 0.82 |
| **TCC** | % Δ/IQR | -6.95 (-21.93, 10.9) | -3.95 (-10.4, 2.5) | 5.18 (-5.5, 17.07) | -3.93 (-10.9, 3.57) | 1.68 (-9.36, 14.07) | -5.60 (-13.01, 8.6) |
|  | p | 0.42 | 0.23 | 0.36 | 0.62 | 0.78 | 0.32 |
| **TCS** | % Δ/IQR | 13.79 (-1.82, 31.87) | 1.44 (-3.89, 6.76) | 7.81 (-3, 19.83) | -0.65 (-6.78, 5.89)^a^ | 0.17 (-9.12, 10.41) ^a^ | 0.12 (-6.88, 12.69) |
|  | p | 0.09* | 0.60 | 0.16 | 0.63 | 0.97 | 0.85 |
| **EPB^b^** | % Δ/IQR | 3.40 (-15.66, 26.78) | -2.28 (-9.73, 5.18) | -0.25 (-11.87, 12.9)^a^ | -0.31 (-49.9, 98.2) | 4.83 (-8.72, 20.39) | -5.05 (-13.8, 4.58) |
|  | p | 0.75 | 0.55 | 0.97 | 0.32 | 0.5 | 0.95 |
| **BPB** | % Δ/IQR | 2.51 (-10.1, 16.9) | -3.47 (-8.18, 1.23) | -3.51 (-12.19, 6.04) | -1.80 (-7.08, 3.79) | -5.69 (-13.47, 2.78) | 4.38 (-4.17, 12.69) |
|  | p | 0.71 | 0.15 | 0.46 | 0.71 | 0.18 | 0.56 |
| **MPB** | % Δ/IQR | 4.27 (-8.72, 19.1) | -5.94 (-10.7, -1.19) | -8.11 (-16.46, 1.09) | -5.33 (-10.6, 0.19) | -7.76 (-15.4, 0.61) ^a^ | 3.14 (-2.95, 9.61) |
|  | p | 0.54 | 0.01** | 0.08* | 0.10 | 0.07* | 0.08* |
| **PPB** | % Δ/IQR | 3.55 (-10.07, 19.22) | -6.32 (-11.35, -1.3) | -7.02 (-15.95, 2.86) | -1.95 (-8.11, 4.62) | -6.35 (-14.9, 3.03) ^a^ | 5.40 (-2.2, 17.62) |
|  | p | 0.63 | 0.01** | 0.16 | 0.54 | 0.18 | 0.57 |
|  | | **24-28 weeks gestation** | | | | | |
|  |  | **CRH** | **SHBG** | **Testosterone** | **Progesterone** | **Estriol** | **Progesterone/Estriol Ratio** |
| **2,4-DCP** | % Δ/IQR | 9.66 (0.67, 19.45) | 2.50 (-3.84, 8.83) | 0.50 (-9.18, 11.22) | 5.81 (-3, 15.42) | 0.53 (-7.55, 9.31) | 5.07 (-2.4, 14.89) |
|  | p | 0.04** | 0.44 | 0.92 | 0.20 | 0.90 | 0.24 |
| **2,5-DCP** | % Δ/IQR | 3.92 (-3.66, 12.09) | 0.22 (-5.35, 5.79) | -0.27 (-8.76, 9.01)^a^ | -0.80 (-8.01, 6.97) | -4.58 (-11.2, 2.56) | 3.74 (-2.69, 11.87) |
|  | p | 0.32 | 0.94 | 0.95 | 0.83 | 0.20 | 0.32 |
| **BPA** | % Δ/IQR | -3.53 (-13.3, 7.32) | -0.48 (-8.26, 7.31) | -17.37 (-26.7, -6.87)^a^ | -3.65 (-13.4, 7.2) | -5.27 (-14.4, 4.87) | 1.86 (-6.16, 14.1) |
|  | p | 0.51 | 0.90 | 0.002** | 0.49 | 0.30 | 0.72 |
| **BPF^b^** | % Δ/IQR | -1.64 (-16.7, 16.08) | -0.30 (-12.5, 11.9) | -10.98 (-24.82, 5.41) | 4.68 (-10.1, 21.88) | 4.57 (-9.89, 21.35) | 0.04 (-13.02, 15.07) |
|  | p | 0.84 | 0.96 | 0.18 | 0.56 | 0.56 | 1.00 |
| **BPS** | % Δ/IQR | -7.61 (-16.3, 2.01) | -1.77 (-9.38, 5.84) | -6.26 (-15.49, 3.98) | -3.85 (-13.1, 6.4) | -2.05 (-10.75, 7.5) | -1.86 (-9.81, 7.49) |
|  | p | 0.12 | 0.65 | 0.22 | 0.45 | 0.66 | 0.69 |
| **BP-3** | % Δ/IQR | 1.71 (-7.23, 11.51) | 1.80 (-5.09, 8.69) | -2.59 (-12.66, 8.64) | 1.29 (-7.62, 11.1) | 3.41 (-5.31, 12.94) | -2.09 (-9.89, 6.96) |
|  | p | 0.72 | 0.61 | 0.64 | 0.79 | 0.46 | 0.63 |
| **TCC** | % Δ/IQR | -5.00 (-16.1, 7.57) | -9.71 (-19.1, -0.27) | 10.25 (-3.14, 25.5) | -1.80 (-13.9, 12.0) | -1.94 (-13.1, 10.7) | 0.14 (-7.77, 15.3) |
|  | p | 0.42 | 0.05** | 0.14 | 0.79 | 0.75 | 0.98 |
| **TCS** | % Δ/IQR | 8.46 (-2.31, 20.43) | 4.27 (-3.44, 11.98) | 12.46 (-0.47, 27.08) | 9.72 (-1.27, 21.9)^a^ | 13.17 (2.34, 25.2) ^a^ | -3.00 (-12.61, 6.43) |
|  | p | 0.13 | 0.28 | 0.06* | 0.09* | 0.02** | 0.56 |
| **EPB^b^** | % Δ/IQR | -3.51 (-17.06, 12.3) | -2.36 (-13.99, 9.3) | 13.05 (-3.38, 32.28)^a^ | -4.05 (-18.03, 12.3) | -11.47 (-23.3, 2.16) | 8.43 (-5.77, 24.76) |
|  | p | 0.29 | 0.69 | 0.13 | 0.61 | 0.10 | 0.26 |
| **BPB** | % Δ/IQR | -6.35 (-13.8, 1.71) | -3.77 (-9.86, 2.31) | -8.88 (-17.24, 0.32) | -2.20 (-10.1, 6.4) | -3.30 (-10.8, 4.87) | 1.05 (-7.26, 8.5) |
|  | p | 0.12 | 0.23 | 0.06* | 0.61 | 0.42 | 0.80 |
| **MPB** | % Δ/IQR | 8.20 (-1.67, 19.06) | 1.25 (-5.82, 8.32) | -7.70 (-17.49, 3.25) | 8.21 (-1.7, 19.11) | 6.54 (-2.86, 16.84) ^a^ | 1.05 (-7.83, 10.78) |
|  | p | 0.11 | 0.73 | 0.16 | 0.11 | 0.18 | 0.82 |
| **PPB** | % Δ/IQR | 8.87 (-1.95, 20.89) | -0.39 (-8.13, 7.35) | -2.21 (-13.55, 10.61) | 9.67 (-1.3, 21.85) | 8.92 (-1.56, 20.52) ^a^ | 0.19 (-8.79, 10.8) |
|  | p | 0.11 | 0.92 | 0.72 | 0.09* | 0.10 | 0.97 |
| 2,4-DCP: 2,4-dichlorophenol; 2,5-DCP: 2,5-dichlorophenol; BP-3: Benzophenone; TCS: Triclosan; TCC: Triclocarban; EPB: ethylparaben; MPB: Methylparaben; BPB: Butylparaben; PPB: Propylparaben  Beta coefficients are transformed into percent change of hormone in an IQR change in the exposure. Beta coefficients and their 95% CI are displayed. ^a^ represents a significant interaction term between the exposure*visit in LMM models (p<0.05). *p values < 0.1. ** p values < 0.05. **^b^** Dichotomous variable. Models adjusted for specific gravity, study visit, body mass index (BMI) at the first study visit, maternal age, the number of hours of second-hand smoking exposure per day, and a socio-economic variable. | | | | | | | |

Supplementary Table 2: Results of the adjusted MLRs regressing thyroid hormones versus exposure biomarkers by visit.

|  | **16-20 weeks gestation** | | | | | |
| --- | --- | --- | --- | --- | --- | --- |
|  |  | **TSH** | **FT4** | **T3** | **T4** | **T3/T4 Ratio** |
| **2,4-DCP** | % Δ/IQR | 6.72 (-2.7, 17.06) | 0.21 (-1.4, 1.82) | -2.22 (-4.55, 0.1) | -0.85 (-3.07, 1.37) | -1.56 (-3.95, 0.84) |
|  | p | 0.17 | 0.80 | 0.06* | 0.45 | 0.20 |
| **2,5-DCP** | % Δ/IQR | 3.01 (-6.3, 13.25) | 0.78 (-0.86, 2.41) | -1.32 (-3.7, 1.06) | 0.51 (-1.76, 2.77) | -2.12 (-4.55, 0.32) |
|  | p | 0.54 | 0.35 | 0.28 | 0.66 | 0.09* |
| **BPA** | % Δ/IQR | -4.08 (-12.94, 5.68) | -1.38 (-3.04, 0.29) | 1.04 (-1.38, 3.47) | 0.19 (-2.1, 2.48) | 0.96 (-1.54, 3.46) |
|  | p | 0.40 | 0.11 | 0.40 | 0.87 | 0.45 |
| **BPF^b^** | % Δ/IQR | 15.44 (-5.92, 41.66) | 6.94 (3.26, 10.62)^a^ | 0.26 (-4.94, 5.45) | 3.00 (-1.87, 7.87) | -2.42 (-8.31, 3.47) |
|  | p | 0.17 | 0.0002** | 0.92 | 0.23 | 0.42 |
| **BPS** | % Δ/IQR | -11.93 (-22.49, 0.07) | 0.32 (-1.99, 2.62) | 2.50 (-0.56, 5.56) | 0.38 (-2.57, 3.33) | 2.69 (-0.74, 6.12) |
|  | p | 0.05* | 0.79 | 0.11 | 0.80 | 0.13 |
| **BP-3** | % Δ/IQR | -5.90 (-14.75, 3.87) | -1.37 (-3.07, 0.33) | -1.10 (-3.57, 1.38) | -1.74 (-4.06, 0.58) | 0.90 (-4.88, 2.77) |
|  | p | 0.23 | 0.12 | 0.39 | 0.14 | 0.49 |
| **TCC** | % Δ/IQR | -10.72 (-24.34, 5.34) | 0.06 (-2.93, 3.04) | 6.27 (2.38, 10.17) | 1.00 (-2.85, 4.85) | 5.67 (1.24, 10.10) |
|  | p | 0.18 | 0.97 | 0.002** | 0.61 | 0.01** |
| **TCS** | % Δ/IQR | 5.49 (-8.15, 21.16) | 0.29 (-2.11, 2.7) | -0.81 (-4.32, 2.69) | -0.25 (-3.55, 3.06) | -0.28 (-3.86, 3.3) |
|  | p | 0.45 | 0.81 | 0.65 | 0.88 | 0.88 |
| **EPB^b^** | % Δ/IQR | -2.50 (-19.56, 18.16) | -0.75 (-4.18, 2.69) | -1.47 (-6.11, 3.17) | -1.02 (-5.45, 3.41) | -1.12 (-6.34, 4.09) |
|  | p | 0.80 | 0.67 | 0.54 | 0.65 | 0.67 |
| **BPB** | % Δ/IQR | -2.58 (-13.87, 10.2) | 0.74 (-1.4, 2.88) | 0.18 (-2.88, 3.25) | 1.24 (-1.68, 4.17) | -1.46 (-4.62, 1.70) |
|  | p | 0.68 | 0.50 | 0.91 | 0.41 | 0.37 |
| **MPB** | % Δ/IQR | -11.69 (-21.97, -0.06) ^a^ | 1.00 (-1.16, 3.16) | 0.33 (-2.8, 3.45) | 1.22 (-1.76, 4.2) | -1.26 (-4.45, 1.94) |
|  | p | 0.05** | 0.36 | 0.84 | 0.42 | 0.44 |
| **PPB** | % Δ/IQR | -9.74 (-20.79, 2.85) | 0.82 (-1.47, 3.11) | 1.51 (-1.78, 4.8) | 1.32 (-1.83, 4.47) | -0.06 (-3.44, 3.32) |
|  | p | 0.12 | 0.48 | 0.37 | 0.41 | 0.97 |
|  |  |  |  |  |  |  |
|  | **24-28 weeks gestation** | | | | | |
|  |  | **TSH** | **FT4** | **T3** | **T4** | **T3/T4 Ratio** |
| **2,4-DCP** | % Δ/IQR | 2.28 (-7.3, 12.84) | 0.53 (-1.4, 2.46) | 0.49 (-2.92, 3.89) | 0.67 (-2.32, 3.66) | -0.73 (-6.4, 4.94) |
|  | p | 0.65 | 0.59 | 0.78 | 0.66 | 0.80 |
| **2,5-DCP** | % Δ/IQR | 0.50 (-7.81, 9.56) | 1.01 (-0.68, 2.7) | 2.12 (-0.86, 5.09) | 0.83 (-1.8, 3.45) | -0.45 (-5.55, 4.65) |
|  | p | 0.91 | 0.24 | 0.16 | 0.54 | 0.86 |
| **BPA** | % Δ/IQR | 2.68 (-9.14, 16.03) | 2.38 (0.04, 4.72) | 4.33 (0.11, 8.55) | 2.12 (-1.62, 5.85) | 1.00 (-5.72, 7.72) |
|  | p | 0.67 | 0.05** | 0.05** | 0.27 | 0.77 |
| **BPF^b^** | % Δ/IQR | 1.23 (-15.77, 21.66) | -2.14 (-5.74, 1.45)^a^ | -1.17 (-7.46, 5.12) | 1.19 (-4.33, 6.70) | 8.39 (-10.62, 27.39) |
|  | p | 0.90 | 0.24 | 0.72 | 0.67 | 0.39 |
| **BPS** | % Δ/IQR | 1.10 (-9.89, 13.43) | -1.40 (-3.75, 0.96) | -1.35 (-5.35, 2.66) | -1.01 (-4.66, 2.63) | -0.68 (-6.28, 4.92) |
|  | p | 0.85 | 0.25 | 0.51 | 0.59 | 0.81 |
| **BP-3** | % Δ/IQR | -5.48 (-15.04, 5.17) | 0.99 (-1.1, 3.08) | -3.11 (-6.78, 0.56) | -1.08 (-4.32, 2.17) | -3.46 (-9.22, 2.3) |
|  | p | 0.30 | 0.35 | 0.10 | 0.52 | 0.24 |
| **TCC** | % Δ/IQR | -7.90 (-20.02, 6.06) | -0.81 (-3.75, 2.13) | 5.64 (0.81, 10.46) | 0.71 (-3.8, 5.22) | 0.41 (-6.79, 7.61) |
|  | p | 0.25 | 0.59 | 0.02** | 0.76 | 0.91 |
| **TCS** | % Δ/IQR | 5.46 (-6.44, 18.86) | -1.20 (-3.54, 1.14) | -5.81 (-9.9, -1.73) | -1.82 (-5.47, 1.82) | -1.33 (-8.32, 5.67) |
|  | p | 0.39 | 0.32 | 0.01** | 0.33 | 0.71 |
| **EPB^b^** | % Δ/IQR | -10.15 (-24.31, 6.66) | 0.39 (-3.19, 3.97) | -1.08 (-7.07, 4.91) | 2.78 (-2.72, 8.27) | 12.88 (-18.25, 44.01) |
|  | p | 0.22 | 0.83 | 0.72 | 0.32 | 0.42 |
| **BPB** | % Δ/IQR | -7.03 (-15.34, 2.11) | 0.65 (-1.2, 2.5) | -0.74 (-4.01, 2.54) | 1.32 (-1.56, 4.2) | -3.45 (-8.78, 1.89) |
|  | p | 0.13 | 0.49 | 0.66 | 0.37 | 0.21 |
| **MPB** | % Δ/IQR | -7.22 (-16.8, 3.47) ^a^ | 0.84 (-1.31, 2.98) | -2.96 (-6.74, 0.81) | 0.95 (-2.4, 4.29) | -4.70 (-10.15, 0.76) |
|  | p | 0.18 | 0.45 | 0.13 | 0.58 | 0.09* |
| **PPB** | % Δ/IQR | -8.89 (-19.14, 2.65) | 1.10 (-1.25, 3.44) | -1.72 (-5.87, 2.44) | 0.76 (-2.9, 4.41) | -6.08 (-12.38, 0.23) |
|  | p | 0.13 | 0.36 | 0.42 | 0.69 | 0.06* |
| 2,4-DCP: 2,4-dichlorophenol; 2,5-DCP: 2,5-dichlorophenol; BP-3: Benzophenone; TCS: Triclosan; TCC: Triclocarban; EPB: ethylparaben; MPB: Methylparaben; BPB: Butylparaben; PPB: Propylparaben  Beta coefficients are transformed into percent change of hormone in an IQR change in the exposure. Beta coefficients and their 95% CI are displayed. ^a^ represents a significant interaction term between the exposure*visit in LMM models (p<0.05). *p values < 0.1. **p values < 0.05. **^b^** Dichotomous variable. Models adjusted for specific gravity, study visit, body mass index (BMI) at the first study visit, maternal age, the number of hours of second-hand smoking exposure per day, and a socio-economic variable. | | | | | | |

|  | **Prog** | | **SHBG** | | **FT4** | | **TSH** | |
| --- | --- | --- | --- | --- | --- | --- | --- | --- |
|  | **Prelim** | **New** | **Prelim** | **New** | **Prelim** | **New** | **Prelim** | **New** |
| **2,4-DCP** |  |  |  |  |  |  |  |  |
| **2,5-DCP** |  |  | 1↓ 3↑ |  |  |  |  |  |
| **BPA** |  |  |  |  | ↑ | 3↑ |  |  |
| **BP-3** |  |  | 3↑ |  |  |  |  |  |
| **TCS** |  | (3↑) |  |  |  |  |  |  |
| **MPB** |  |  | ↑ | 1↓ | ↑ |  |  | 1↓ |
| **BPB** |  |  |  | ↓ | ↑ |  |  |  |
| **PPB** |  | (3↑) |  | 1↓ |  |  |  |  |
| 2,4-DCP: 2,4-dichlorophenol; 2,5-DCP: 2,5-dichlorophenol; BP-3: Benzophenone; TCS: Triclosan; MPB: Methylparaben; BPB: Butylparaben; PPB: Propylparaben ***** Previous analysis: Aker AM, Watkins DJ, Johns LE, Ferguson KK, Soldin OP, Anzalota Del Toro LV, et al. Phenols and parabens in relation to reproductive and thyroid hormones in pregnant women. Environ Res. 2016;151:30–7. Numbers in cell refer to a significant association observed in stated study visit only. Associations in brackets refer to suggestive associations with p values between 0.05 and 0.10. | | | | | | | | |

Supplementary Table 3: Result comparison between the common exposure biomarkers and hormones from the preliminary analyses previously published* and the current analysis
