# Supplementary material for: A repeated measures study of phenol, paraben and Triclocarban urinary biomarkers and circulating maternal hormones during gestation in the Puerto Rico PROTECT cohort
Source: Environ Health. 2019 Apr 2;18:28. doi: 10.1186/s12940-019-0459-5 (PMC6444601; doi:10.1186/s12940-019-0459-5)
Supplement: Supplementary file 2 — Adjusted multiple linear regressions of hormones versus urinary concentrations of biomarkers stratified by study visit. Visit 1: 16-20 weeks; Visit 3: 24-28 weeks. EPB and BPF are categorical variables. * represents at least one marginal association between the urinary concentration and the hormone across the four time points. ** represents at least one significant association between the urinary biomarker concentration and the hormone across the four time points. BPF and EPB were dichotomous variables. 2,4-DCP: 2,4-dichlorophenol; 2,5-DCP: 2,5-dichlorophenol; BP-3: Benzophenone; TCS: Triclosan; TCC: Triclocarban; EPB: ethylparaben; MPB: Methylparaben; BPB: Butylparaben; PPB: Propylparaben (DOCX 664 kb) [file 12940_2019_459_MOESM2_ESM.docx]

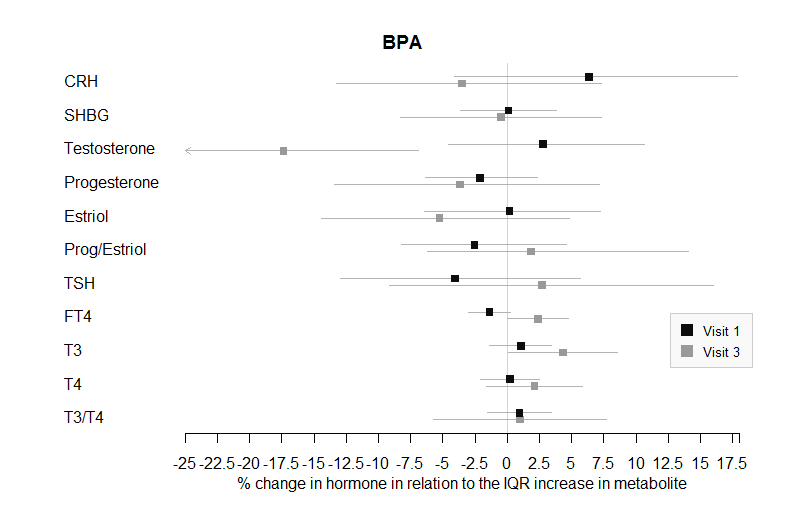

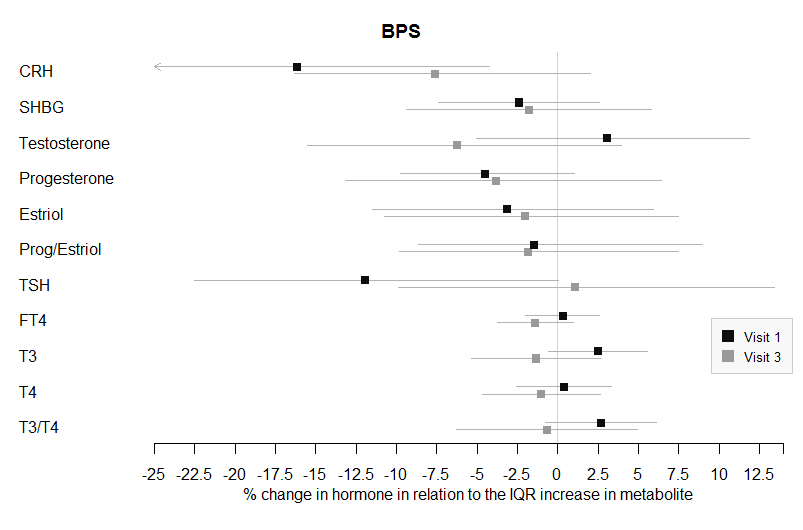

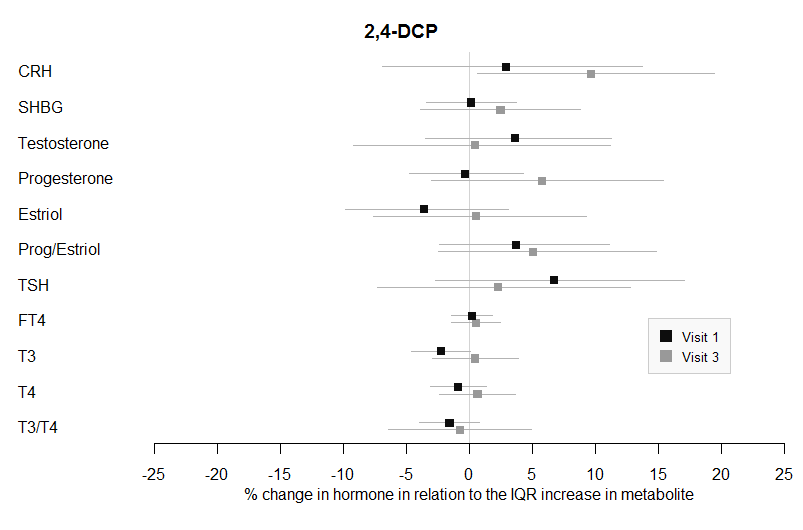


*

*

**

*

**

**


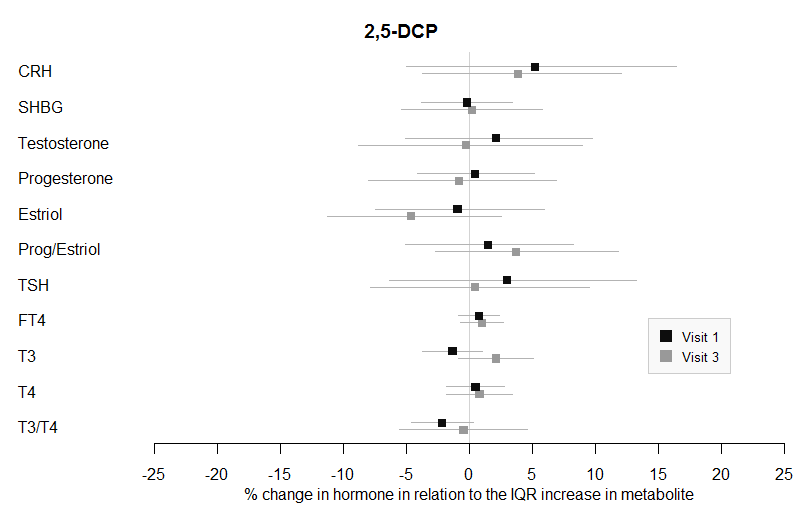

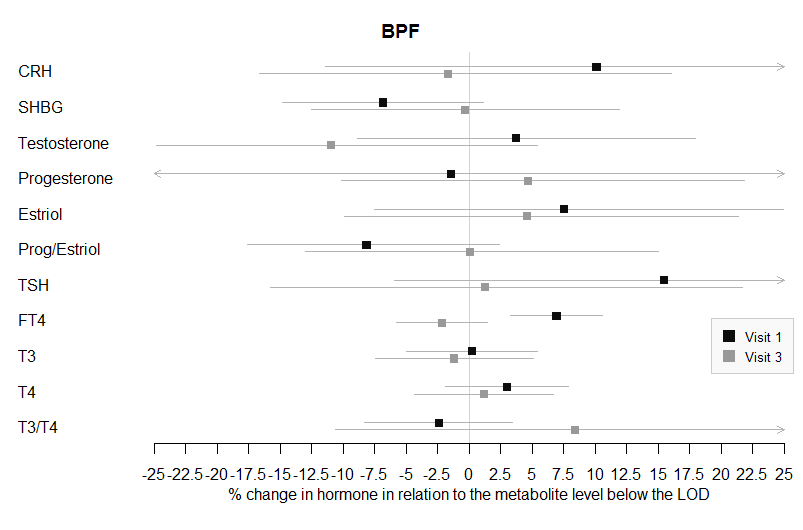

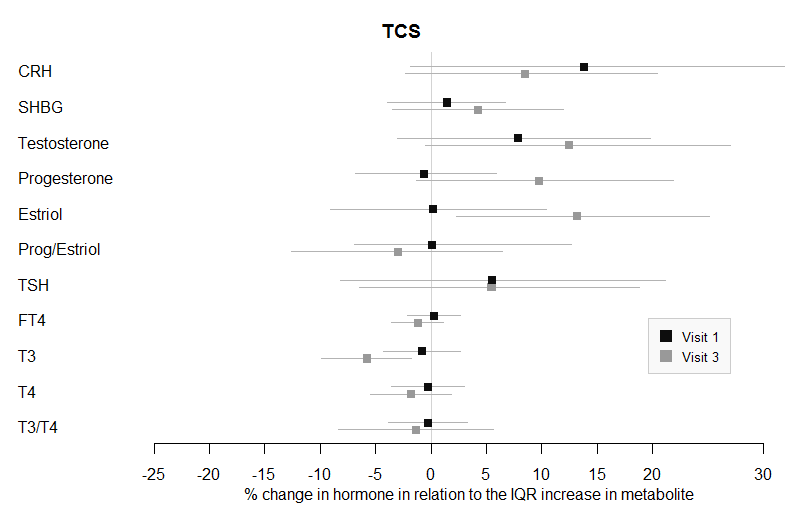

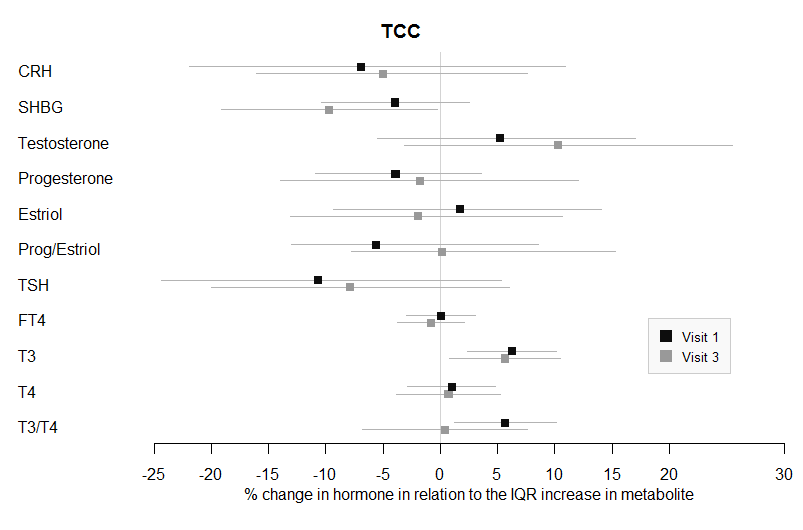


**

*

**

*

**

**

**

**


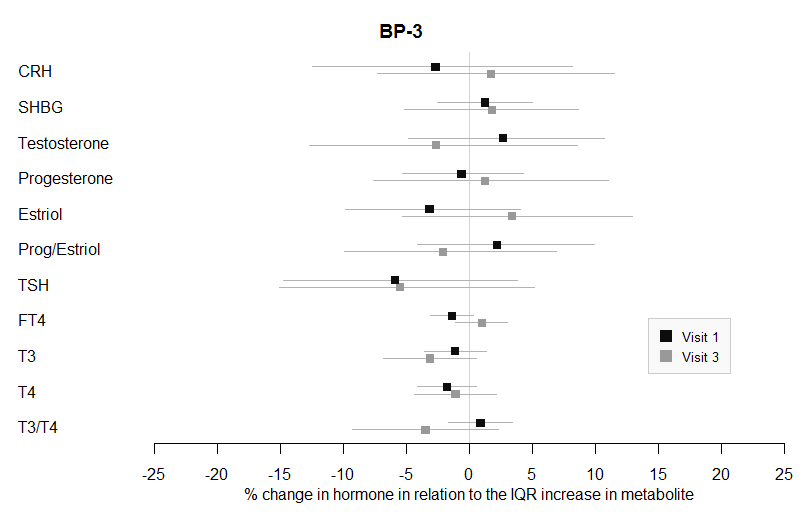

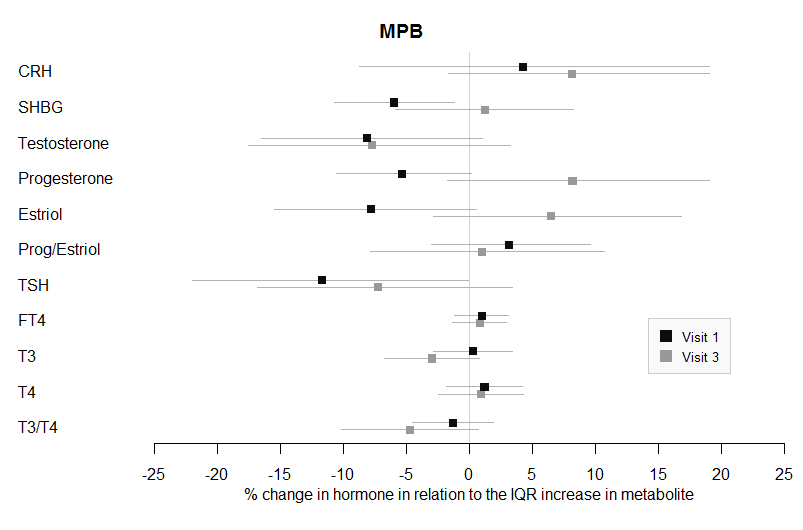

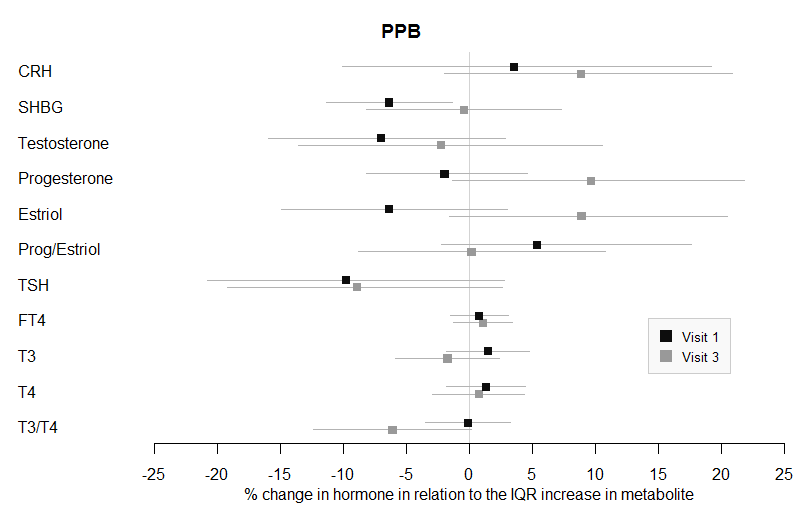


**

**

*

*

*

**

*

*

*


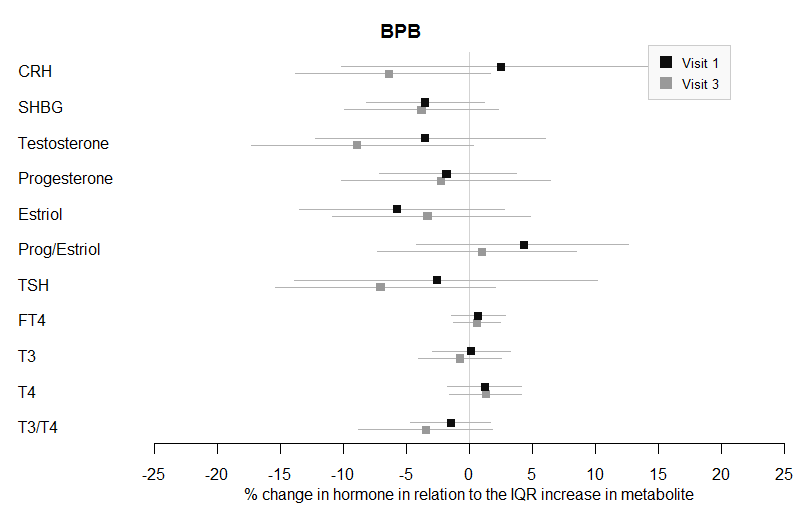

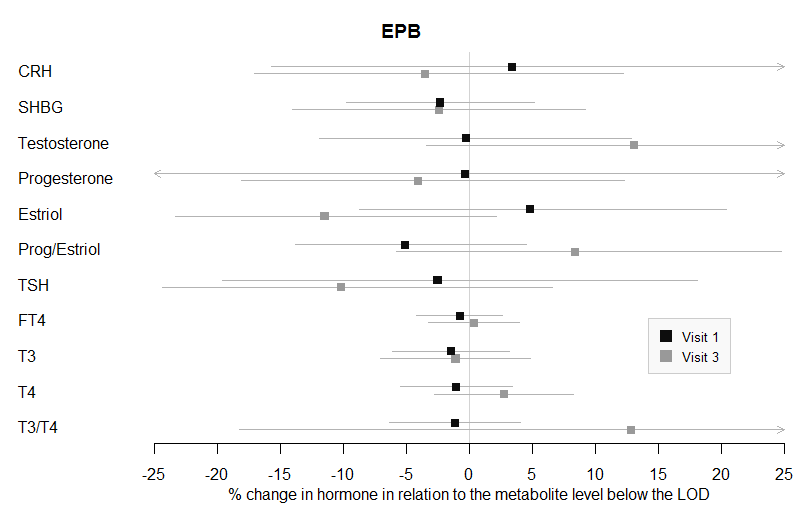


Figure 2: Adjusted multiple linear regressions of hormones versus urinary concentrations of biomarkers stratified by study visit. Visit 1: 16-20 weeks; Visit 3: 24-28 weeks. EPB and BPF are categorical variables. * represents at least one marginal association between the urinary concentration and the hormone across the four time points. ** represents at least one significant association between the urinary biomarker concentration and the hormone across the four time points. BPF and EPB were dichotomous variables. 2,4-DCP: 2,4-dichlorophenol; 2,5-DCP: 2,5-dichlorophenol; BP-3: Benzophenone; TCS: Triclosan; TCC: Triclocarban; EPB: ethylparaben; MPB: Methylparaben; BPB: Butylparaben; PPB: Propylparaben
